# Supplementary material for: pH Effect on the Structure, Rheology, and Electrospinning of Maize Zein
Source: Foods. 2023 Mar 25;12(7):1395. doi: 10.3390/foods12071395 (PMC10093575; doi:10.3390/foods12071395)
Supplement: Supplementary file 1 [file foods-12-01395-s001.zip › foods-2233128-supplementary.pdf]

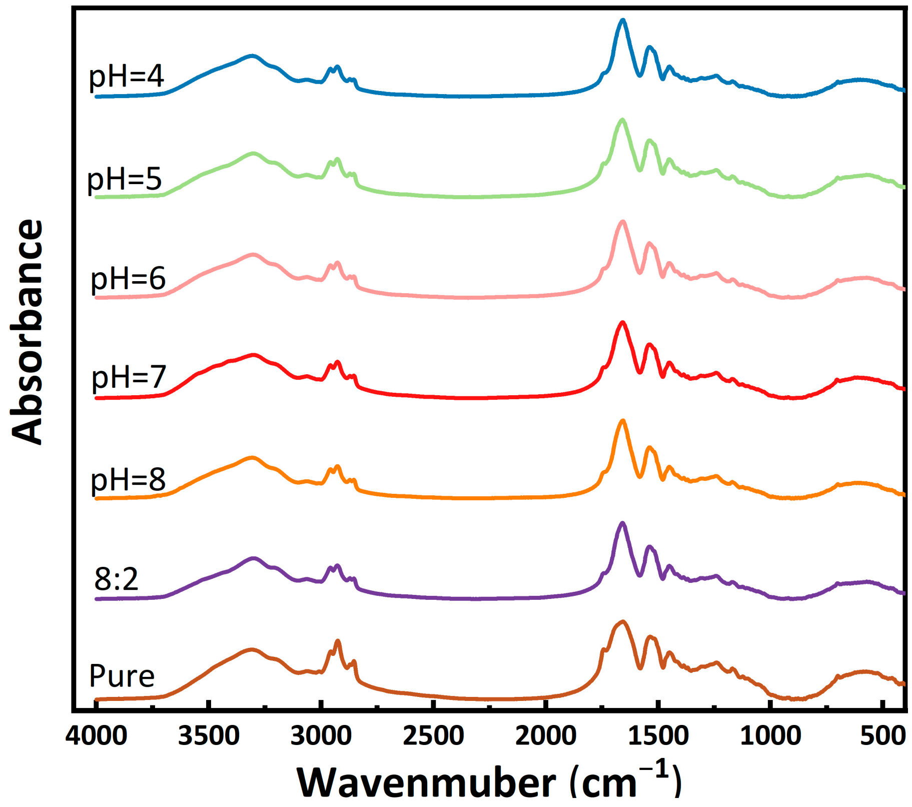

Figure S1. The FT-IR spectra of zein powder and zein film obtained by zein solutions with different pH values.
